# “A source of empowerment and well-being”: Experiences of a dance and yoga intervention for young girls with functional abdominal pain disorders

**DOI:** 10.3389/fped.2023.1040713

**Published:** 2023-04-21

**Authors:** Sofie Högström, Mats Eriksson, Evalotte Mörelius, Anna Duberg

**Affiliations:** ^1^ University Health Care Research Center, Faculty of Medicine and Health, Örebro University, Örebro, Sweden; ^2^ Faculty of Medicine and Health, School of Health Sciences, Örebro University, Örebro, Sweden; ^3^ Edith Cowan University, School of Nursing and Midwifery, Joondalup, WA, Australia; ^4^ Department of Health, Medicine and Caring Sciences, Linköping University, Linköping, Sweden

**Keywords:** dance, experiences, functional abdominal pain disorders, intervention, yoga 3(20)

## Abstract

**Background:**

Functional abdominal pain disorders are common among children and adolescents worldwide and effective treatments are needed to alleviate suffering for these children and their families. This study aimed to explore the experience of participating in a combined dance and yoga intervention from the perspectives of girls aged 9–13 years with functional abdominal pain disorders.

**Materials and Methods:**

A randomized controlled trial called Just in TIME (*Try, Identify, Move and Enjoy*) recruited 121 girls aged 9–13 years with functional abdominal pain disorders. The eight-month intervention combined dance and yoga twice a week, focusing on enjoyment, socialization and playful creativity in an undemanding and non-judgemental environment. The intervention group comprised 64 girls, of whom 25 were purposefully selected for this qualitative interview study. Semi-structured interviews were conducted and analysed using qualitative content analysis with an inductive approach.

**Results:**

The girls' experiences of the Just in TIME intervention can be described as “A source of empowerment and well-being which facilitated personal growth and new ways of engaging in life”. The main category was derived from six generic categories: “A sense of belonging”, “Joy and emotional expression through movement”, “Relief from pain”, “More self-confident”, “More active in daily life” and “A sense of calm.”

**Conclusions:**

Regular participation in an eight-month intervention with combined dance and yoga in a supportive and non-judgemental atmosphere can ease pain and strengthen inner resources, resulting in empowerment, well-being and a more active life for girls with functional abdominal pain disorders.

**Trial registration:**

The Just in TIME study is available online at clinicaltrials.gov, ID: NCT02920268.

## 1. Introduction

Functional abdominal pain disorders (FAPDs) affect children globally, with a pooled prevalence of 13.5%, and they are more common among girls than boys (1). According to the ROME IV criteria, FAPDs are classified as Functional Dyspepsia, Irritable Bowel Syndrome (IBS), Abdominal Migraine, and Functional Abdominal Pain—Not Otherwise Specified (2). Biological, psychological and psychosocial factors contribute to the development and maintenance of this complex disorder (3–5), which is explained as a dysregulation of the gut–brain axis (4, 6). This axis is understood as a bidirectional communication system including neural, endocrine, immune and metabolic pathways between the gut and the brain (4). The main symptom of FAPDs is abdominal pain (7) and it is associated with anxiety, depression (8, 9), low quality of life (8–10) and school absenteeism among affected children (9, 11). The psychological factors can be both a consequence and part of the cause of FAPDs, but psychological factors alone are not proven sufficient to cause FAPDs (4). In addition, somatization is an important predictor of outcomes in children with FAPDs (12), and one study on children with IBS found that the effect of anxiety and depression on abdominal pain can be mediated by somatization and catastrophizing pain (13). It is noteworthy that abdominal pain in childhood has been shown to be a predictor of anxiety disorders (14) and severe mental illness in adulthood (15).

There is some evidence for dietary treatment (16, 17) and pharmacological treatment (18) in children with FAPDs. Probiotics seem to show promise for reducing pain in children with IBS, but the evidence is not sufficient (19). Studies have shown that cognitive behavioural therapy and hypnotherapy can reduce pain in children with FAPDs (4, 20, 21) and could be considered as treatments (21). However, more well-designed studies are needed to establish effective treatments for this patient group (7, 20, 22, 23).

Yoga is a practice that involves physical postures, conscious regulation of breathing and attention-based techniques (24). Yoga-based practices have shown promising potential to reduce pain intensity, decrease school absenteeism (25), reduce IBS symptoms and improve physical functioning and quality of life for children with FAPDs (26). But due to limitations in study methods (26), risk of bias and high imprecision (21), no recommendations have been made for yoga as a routine treatment for children with FAPDs, and more well-designed studies of high quality are needed (21, 26).

Several studies with children and adolescents emphasize that dance intervention can improve both psychological (27–30) and physiological (30, 31) health, and in children aged 9–13 years with FAPDs, the Just in TIME (*Try, Identify, Move and Enjoy*) intervention with combined dance and yoga was shown to reduce abdominal pain (32). Qualitative studies exploring experiences of interventions among children with FAPDs are few and this is, to our knowledge, the first study to evaluate their experiences of a dance and yoga intervention for children with FAPDs. A qualitative study can provide insights about the intervention from the participants’ perspective to deepen understanding in the field of non-pharmacological interventions for children with FAPDs. The aim of this study was to explore experiences of participating in a dance and yoga intervention from the perspectives of girls aged 9–13 years with FAPDs.

## 2. Materials and methods

### 2.1. Design

This study was a qualitative, descriptive study utilizing content analysis with an inductive approach according to Elo and Kyngäs (33).

### 2.2. Participants and setting

This study was part of a larger randomized controlled trial involving 121 girls aged 9–13 years with FAPDs (34). The 64 girls in the intervention group participated in dance and yoga sessions twice weekly for eight months and the 57 girls in the control group were encouraged to live as usual. Both groups had access to standard health care when needed. The first year of recruitment was restricted to girls aged 9 to 12 years at one study site in a middle-sized city in Sweden. In the second and third year of recruitment, the upper age limit was raised to 13 years and one more study site in another middle-sized city was added to broaden the study sample. Participants were recruited from outpatient clinics at the university hospital pediatrics departments, as well as from primary health care, school health services and the general public (from one site only in the first year and from both sites in the second and third year). We also recruited from a counselling unit for children and adolescents in one of the study-sites. The participants were recruited through advertisements and through information letters sent or given to families with girls in the target age range who had visited the outpatient clinics at the university hospital pediatrics departments because of FAP, IBS or constipation or visited the clinics for the first time. The inclusion criteria for the study were as follows: Girls aged 9–13 years, diagnosed with IBS and/or functional abdominal pain according to the ROME III criteria (35), which were current at that time, and persistent pain after examination at the paediatric clinic. The exclusion criteria were: co-existing celiac disease or inflammatory bowel disease, difficulty following oral instructions (such as hearing impairment, mental disability or language difficulty), simultaneous treatment with cognitive behavioural therapy and severe depression for which other treatment was needed (34). The Children's Depression Screener (ChilD-S) (36) was used to measure depression, and girls who scored 13 or higher were offered contact with a psychologist to assess whether they needed further support and whether they could participate in the study.

The Just in TIME intervention (hereafter referred to as JiT) combined dance and yoga in each session, which consisted of 30 min of dance, 25 min of yoga (including relaxation) and 5 min of short reflection. The intervention, designed by one of the authors (AD), aimed to appeal to the target age group regarding choice of music, exercises and choreographies. It was underpinned by the principals of the self-determination theory (SDT), which suggests that self-motivation, personal development and well-being are enhanced when the basic needs of competence, autonomy and relatedness are satisfied (37). A recent meta-analysis showed that interventions based on SDT led to improvements on indices of health and STD constructs (38). The JiT intervention was based on the theory of embodiment, which implies that the motor system and the cognitive affective system are intertwined and therefore influence each other (39–41). Each dance and yoga group consisted of between 7 and 14 girls and was led by a trained dance and yoga instructor (or sometimes two instructors together), who also had previous experience of working with children. Throughout all sessions the focus was on enjoyment, socialization and playful creativity in an undemanding environment. The dance choreographies were mostly guided by the instructor but also included creative elements where free movement was encouraged. Yoga poses were performed individually, in pairs and in the group (often in a storytelling context) and always ended with a guided relaxation including a brief massage (34). During the eight-month intervention, five participants attended to 90%–100% of the total number of JiT-sessions, thirty-five attended to 50–90% of the sessions, fifteen attended to 10%–50% of the sessions and nine participants attended to less than 10% of the sessions.Twenty-five participants from the intervention group were purposefully selected (42) for the interview study. Sampling was made at three different time points (after the intervention was completed in study year 1, 2 and 3). Ten participants were selected for interviews the first year, eleven the second year and four in the third year. The selection was based on regular participation during the eight-month intervention and aimed at variation in the girls' age, level of pain at baseline and participation rate. Phone calls were made to the legal guardians of the eligible girls to invite them to participate in an interview. All but one of the parents agreed to their daughter's participation in the study; they were then sent an information letter and consent forms. Both the legal guardians and participants gave written consent for the interview study. There was a mixture of family backgrounds: some girls lived with both legal guardians all the time, some lived alternating weeks with each legal guardian and some lived with one legal guardian all the time. A majority of the legal guardians worked or studied. Most of the girls had one or two siblings. The highest pain level at baseline ranged from 4 to 10, measured with the Faces Pain Scale—Revised (FPS-R), which is scored from 0 to 10 (43), in a pain diary. The participation rate ranged from 48% to 96.2% attendance at the dance and yoga sessions. See Table 1 for the sample characteristics.

**Table 1:** Characteristics of the sample (*n* = 25).

Characteristics	
Age at inclusion, mean (SD)	10.4 (1.4)
*Highest pain level at baseline, median (IQR)	6 (6-8.5)
Proportion (%) of attendance at the intervention sessions, median (IQR)	75 (65.7-88.3)
Diagnosis, *n* (%) Irritable bowel syndrome Functional abdominal pain	6 (24) 19 (76)

* Measured with the Faces Pain Scale - Revised (FPS-R), scored 0–10 [45]. (IQR = interquartile range.)

### 2.3. Data collection

Semi-structured interviews were conducted within a few weeks after each intervention was completed. Interviews took place in a room at the university research centre in the cities where the study was conducted or at the participant's home, according to their preference. An interview guide with 12 open-ended questions was used, see Table 2. The questions concerned how the participants experienced being part of the dance and yoga intervention, whether it had been useful to them, their current health and their plans for the immediate future.

**Table 2:** Interview guide.

1. How did you come to join this project? 2. Please tell me, what has it been like to participate? 3. What did you like most about the dance and yoga practice?/What did you think was less good? 4. How does your body feel when you are dancing? 5. How does your body feel when you are doing yoga? 6. How did it feel to be part of a group? 7. Have you learned something new in the dance and yoga group that has been useful? If so, what? 8. Have you been able to use anything you have learned, at school or at home? 9. What do you do when you want to relax? 10. How do you feel nowadays? Has it changed compared to last autumn? 11. It will soon be the summer holidays. When the autumn term starts again, what do you think you want to do in your spare time? 12. Is there anything more you want to tell me?

All participants were encouraged to speak freely, and follow-up questions were used to gain rich interview data (44, 45). The interview guide was not pilot tested, and repeated interviews were not used. One legal guardian (in some cases two legal guardians) accompanied the girls to the interviews, and participated for a few minutes at the beginning and end of the interview, but the girls were mainly interviewed separately. Before the interviews started, participants and their legal guardians were informed about the purpose of the study, assured of confidentiality and informed that they were free to terminate the interview at any time. The interviews lasted between 29 and 53 min and were digitally recorded. One of three interviewers conducted each interview. Interviewer 1 (AD) holds a PhD in health sciences, is a registered physiotherapist within child and adolescent psychiatry and is the Just in TIME study intervention coordinator. She has previous experience of qualitative research and had the overall responsibility for the data collection, including supervision of interviewer 2 and 3 regarding interview technique. Interviewer 2 is a medical doctor, (medical student at the time of the interviews) and yoga teacher. Interviewer 3 is a registered physiotherapist and dance instructor for all ages. Both interviewer 2 and 3 worked in the JiT research project at that time and were trained in the intervention method. The participating girls were familiar with the interviewers from information meetings and data collections, and two of the interviewers had been stand-in instructors for some of the intervention sessions.

### 2.4. Data analysis

An external company, with experience in the field, transcribed the interview recordings verbatim. Qualitative content analysis with an inductive approach described by Elo and Kyngäs (33) was used to analyse the interviews. The first author (SH) listened to all of the recordings, and all of the authors read the interview transcripts, writing notes in the margins in an open coding process. SH generated codes by extracting meaning units and condensing them to codes, and then sorted data by content into groups using the NVivo software (46). All authors met and discussed the codes and the content of the interviews. SH continued with the coding and generated subcategories, which aimed to mirror the core content in the interviews. The subcategories resulted in six generic categories through abstraction. This process included going back and forth through the different steps. All authors discussed the subcategories and generic categories several times during the analysis phase, and SH and AD discussed the coding item by item. An example of the analysis process is presented in Table 3.

**Table 3:** Example of the analysis process.

Meaning unit	Code	Subcategory	Generic category
Now that I have like built up my self-confidence I feel like I dare to do more, I dare to speak in front of the class which I didn’t before. Because then I had bad stomach pain and I feel like this has really improved. And I think that is really nice.	I dare to do more now when my confidence is better	More confident in myself	More self-confident

The main category was identified through abstraction from the generic categories.

## 3. Results

The girl's experiences of the dance and yoga intervention are described by the main category, “*A source of empowerment and well-being which facilitated personal growth and new ways of engaging in life*”. The main category was derived from six generic categories, see Table 4.

**Table 4:** The girls’ experiences of the just in TIME dance and yoga intervention, in six generic categories and one main category.

Generic categories	Main category
A sense of belonging	A source of empowerment and well-being which facilitated personal growth and new ways of engaging in life
Joy and emotional expression through movement	
Relief from pain	
More self-confident	
More active in daily life	
A sense of calm	

The main understanding of the girls' experiences of the JiT intervention was that the empowerment and well-being they experienced during the JiT sessions also influenced them as a person and affected other areas of their lives. The girls felt empowered in several ways: able to handle pain and stressful situations, claiming more space to make their voice heard when speaking up for themselves and others, making new social connections with others and being more active in their own life. A great source of well-being for the girls was the relief from abdominal pain but well-being was also experienced in more ways. They found themselves accepted for who they were, realized that they were not alone with problems related to FAPDs, felt able to practice dance and yoga without performance requirements, and they experienced relief from negative thoughts, pain and anxiety. The generic categories are described one by one in the following sections.

### 3.1. A sense of belonging

The girls found new friends at the JiT intervention and realized that they were not alone with their problems related to FAPDs. They felt support both from instructors and from each other, and they developed a safe community where it was accepted that everybody could be themselves, thus creating *a sense of belonging*.

The atmosphere in the group was hesitant and tense in the beginning but developed over time into a safe community. One girl commented that she felt safe when she knew what was going to happen and who everybody was. The intervention sessions had the same format and were thereby predictable, which contributed to a feeling of safety for her. The girls described group and pair exercises as valuable elements in the process of getting to know each other and coming together as a group. Some expressed the view that the girls-only environment felt safe and their experience was that everybody was kind and including towards each other. The instructors were described as supportive, understanding, kind and encouraging.

*“I think everyone in the group felt that, yes, the cohesion was very good. This is how it was, we worked at good bonding from the very first time and then it got much better. And it was fun to go there.”—Y*

When the girls met, they realized they were not alone in having problems related to FAPDs which was expressed as a comfort to know.

*“It was nice to just, get to be there, you know, to know that everybody has the same problem as me, then you feel more like everybody else.”—G*

They felt that the JiT intervention provided a context where they could be themselves and be accepted for who they were without judgement.

*“When I was here, I felt that I got a place where I was really allowed to be myself and I got to do something I think is fun.” -B*

It meant a lot to the girls that being part of the intervention gave them a chance to meet other girls and make new friends. It was an opportunity to get to know people they might not have met otherwise and some interviewees mentioned this as the best part of the intervention. Some built a friendship that lasted beyond the intervention.

*“That has been the best part, to get real friends for life that you can trust 100%.”*

### 3.2. Joy and emotional expression through movement

The girls experienced dance and yoga as fun and enjoyed the combination. They observed that dance affected their mood and brought out a sense of happiness. They also reported that music and movement gave them an opportunity to express a range of emotions without having to put them in words. These experiences were understood as *joy and emotional expression through movement*.

Dance and yoga—both separately and the combination of these two modalities—were described by the girls as fun, which enhanced their motivation to continue to participate in the intervention, although a few interviewees reported a loss of motivation near the end of the intervention. Some highlighted yoga and some dance as most fun, and the combination offered a variation which they appreciated. Sometimes they found it a struggle to get to the dance and yoga sessions, for several reasons, including abdominal pain, lack of time or tiredness after school, but they felt it was worth the effort since it felt fine and was fun when they got there. They expressed a sense of joy in being creatively involved in the intervention, for example making up their own variations of movements during yoga storytelling and creating their own improvised dance moves.

*“But like, everybody got to make suggestions and then you, you, might know what a bear looks like, but you got to make up your own bear or your own… boat or something, so it was great fun”—X*

Dance was frequently described as bringing out positive feelings such as happiness and joy. An experience of “opening up” emerged, both as a bodily expression and as a lively, bubbly emotion.

*“It felt like this…”Here I am” like “Now I am coming”… I don't know. It is just a sense of something opening up, and then I become happy and a bit bubbly like this “hee hee hee”.”—M*

Moreover, the girls pointed out that they experienced the different modalities of dance and music as an opportunity to express other emotions such as power, anger and sadness. Some felt that it was a relief to express these emotions without having to put them in words.

*“No but when you have anger inside you, then you have to be able to let it out, because otherwise there will be like a knot in your stomach that you have to get rid of and then you might be rude to your friends. That's why I feel it is pretty nice to dance it away”—C*

### 3.3. Relief from pain

The girls reported that they experienced fewer symptoms of abdominal pain during the JiT sessions and in daily life. They had started to use breathing techniques, relaxation and movements they had learned at the JiT intervention as strategies to manage and reduce abdominal pain in daily life. They also experienced fewer headaches and other somatic pain symptoms. These narratives reflected experiences of *relief from pain*.

If the girls had abdominal pain on a day when they went to JiT in the afternoon, they described an immediate reduction in the pain during the dance and yoga sessions. Some felt relief from pain when dancing and some during yoga and relaxation. One girl explained that she felt relief from pain because of the distraction and shift of focus away from her pain.

“*Yes, it actually disappeared, when I got home there was only a tiny bit of pain left. So I think it is that you think it away, like freeze it out, like “bye!” [giggles].”*

After participating in the JiT sessions for a period of time, the girls experienced an overall reduction in abdominal pain in daily life compared to before the intervention. The abdominal pain decreased both in intensity and frequency; at the time of the interview, some girls no longer suffered from abdominal pain at all. Many of the girls had had issues with recurrent abdominal pain for many years and expressed great relief at finding that their pain had decreased or disappeared lately. One girl described it as a sense of being released from a cage.

“*It is just much nicer when you don’t have stomach pain because you feel free, kind of. It feels like you have been sitting in a cage. And just then, you are released.”—M*

Some girls also described a lessening of other somatic pain symptoms, such as headache or pain in the knee, neck or arm.

Many of the girls started to use exercises from JiT as strategies for managing and reducing abdominal pain in daily life. By developing a deeper awareness of their bodily reactions in connection with different elements in the intervention, they gained new pain relief techniques that they could use when needed. The exercises used most frequently for this were deep breathing and the seated flexion/extension of the spine (seated cat/cow pose, below referred to as “the sea turtle”).

“*And then sometimes, when I get a little abdominal pain I usually do the sea turtle and then it usually disappears”—E*

### 3.4. More self-confident

The girls felt more confident in themselves because they experienced that JiT gave them the courage to claim more space in different social contexts, to care less what other people might think of them and to set boundaries. In particular, they found that they had developed a more accepting attitude toward themselves and an awareness of their body and its needs.

“*Just in TIME has helped me see that I am as I am, here am I, not someone else.”—J*

The girls claimed more space in different contexts. For example, in school: talking more in front of the class, interacting more actively with classmates and raising their hand to answer questions more often than before. Moreover, they experienced a newfound confidence to open up and talk to adults such as teachers, the principal and parents.

“*I have gained more confidence in myself and so I can go and tell adults more now than I could before. If something happens that I find difficult I can talk to some teacher, the principal…yes.”—A*

The girls commented that they cared less in general about what others might think of them in different situations. One girl described how she changed from dancing because she wanted to look cool to others, which she had done previously, to dancing just for fun. She no longer felt the same need for attention from others.

“*I don’t care as much what others think of me, that I must have everybody's attention, to do cool things and all that, no, it's changed, I’ve become more ‘I hang out with whoever I want, I don’t hang out with those who are mean and so on.’ There are some girls in my class that do things that aren’t allowed and I used to hang out with them to be cool but now it is like ‘No, I don’t because I am already cool, I am cool the way I am’, and if others don’t like me for who I am, yeah but then I don’t have to care about that.”—E*

The girls stood up for themselves and for others by speaking up, with a newfound courage, in situations where they or others were mistreated and where they had remained quiet before.

They also seemed to feel that participation in JiT led to an increased acceptance of the fact that it is okay to not appreciate or do what others do. An enhanced inner strength to be oneself.

“*And I don’t do this, I don’t like to talk during the breaks, I rather jump around and do a little bit of this and that, run around instead of standing and talking like the others. So I am a little different and so then I can’t really be myself completely but I have learned that through it [JiT] to be myself anyway.”—B*

Their experience was that physical movement felt good for their body and for their overall sense of well-being. They described their bodies as softer, calmer, stronger and more flexible. One girl expressed her thoughts about what she perceived as good about dance, and she connected it with how it made her feel inside.

“*When you dance you gain more muscles and then you see that, well I might be a bit stronger now and then it feels better inside.”—J*

### 3.5. More active in daily life

Many of the narratives highlighted a sense of being happier and more alert and participating in more daily activities compared to before the intervention. Examples such as an increased attendance in school and seeing friends more often than before emerged, which indicated that the girls were *more active in daily life*.

The girls described an overall sense of being happier and more alert on a daily basis than they were before. One girl emphasized that the biggest difference she felt compared to before the intervention was a stronger sense of happiness. They also remarked that they had gained positive thinking skills, especially in school.

“*Thus to…think more positive, because it makes me, … we had a more positive mindset when we were there. And then I really notice in school that I really think more positively.”—A*

Many of the girls had a history of missing many days of school because of abdominal pain. During the intervention period, their absence from school due to abdominal pain decreased and they managed to spend more time in school instead of going home before the end of the day.

“*It's been fun. And I feel a big difference. Because before I had really bad stomach pain and often stayed at home from school. But now I am almost never off school because of stomach pain”—E*

They also found it easier to concentrate and stay focused during school lessons, which facilitated their schoolwork.

“*If you’re sitting still you can’t focus well when you have stomach pain, but when you aren’t in pain you can focus and then do better in your math exercise book. Then you’re more focused on what you’re doing and it goes faster and stuff like that and then you get more of it right.”—U*

They spent more time with friends now than before when they often choose to stay at home and rest because of their abdominal pain. They also described themselves as being more including towards friends, letting more people join in. One girl observed that she had found new friends, which she thought was because she had been become kinder since she started in the JiT intervention. They felt that they had better endurance to engage in family activities and spent more social time with their family doing things together; for some, this facilitated a closer relationship with their family members. The girls also appreciated the fact that the JiT intervention offered a regular after-school activity, since several of them did not participate in any regular activity at the time of their enrolment in this study.

“ *So now I can do it, much more because before I couldn’t manage it because I had so much pain, like this: no not now because I have stomach pain. You just want to lie down and rest. Now I engage in more activities, for example go outside and also see my friends a lot.”—M*

### 3.6. A sense of calm

The girls experienced the absence of performance requirements in JiT as a relief, that participating in the intervention helped them to let go of other thoughts and they experienced a sense of calm during yoga and relaxation. Their narratives revealed that they now had better sleep, less anxiety and an overall sense of more calmness in daily life, sometimes using exercises from JiT as a strategy to achieve calm. Altogether, these experiences were interpreted as indicating that they had gained *A sense of calm*.

Attending the JiT sessions helped the girls to let go of other thoughts. One girl felt that participation in the intervention brought lightness to her thoughts, which in turn out-competed negative thoughts.

“*They come out. The brain empties and there is space for new thoughts. Wonderful thoughts come in and all dark thoughts disappear.”—Z*

Furthermore, although they sometimes found it hard to explain in words, they expressed appreciation for practicing dance and yoga without any performance requirements. The absence of “right and wrong” meant that they felt less pressure and more sensitivity to their own needs when practicing the movements.

“*It is kind of special in a way but still not that special, … and yet it is. You get to feel what is best for yourself but you still have, like, “this is how you do it’. Yes, when you go to a usual dance, like disco, you have to, like, practise and do it right, right, right, and that is kind of hard. But when you come [to Just in TIME sessions], it just feels more, you get more freedom in the dance. Although there is freedom in all dances, sort of, but it feels much more so right there. It's really hard to explain.”—M*

The girls described how yoga and relaxation felt unfamiliar in the beginning but turned into something that brought a sense of calm and felt comfortable. When they became more familiar with the postures and relaxation techniques, they gained an immediate sense of reduced stress and anxiety during the yoga and relaxation. They highlighted the brief massage during relaxation as something comfortable that strengthened a sense of security and calmness, and many of the girls found the relaxation to be the best part of the yoga sessions. One girl experienced the sessions as too slow with relaxation, which was sometimes challenging for her.

“*The second time was easier I suppose, and the third time J managed to fall asleep and then I realized; yes it's okay, it's okay, it's possible to take it easy. And the fifth time I remember I fell asleep and somewhere there I found ‘yes, they're relaxing, so we can do it too, it got calmer and in the end it had become a part of daily life to just lie down and breathe out and let go.”*

They described an overall sense of increased calmness in several areas of daily life compared to before and commented that they did not worry about various things as much as they used to.

“*I am much calmer everywhere now, home, in school and everywhere”—H*

By using yoga exercises, including relaxation, in situations where they needed to become calm in daily life, they could for example avoid arguments with siblings, manage stressful events and fall asleep more easily at bedtime.

“*Yes I usually, I find it easier to fall asleep too, because I use relaxation techniques we got and suchlike, and then it has been easier for me to let go of everything that happens during the day and I can fall asleep.”—J*

Their increased calmness led to better night-time sleep with less episodes of waking up.

## 4. Discussion

The aim of this study was to explore the experience of an eight-month dance and yoga intervention from the perspectives of girls with FAPDs to deepen understanding in the field of non-pharmacological interventions for this target group. The experiences of the JiT intervention were interpreted in the main category as *a source of empowerment and well-being, which facilitated personal growth and new ways of engaging in life*. The main category was derived from six generic categories: “A sense of belonging”, “Joy and emotional expression through movement”, “Relief from pain”, “More self-confident”, “More active in daily life” and “A sense of calm”. The results indicate that these girls found empowerment and well-being from both the physical activities of combined dance and yoga and from the group setting, with its accepting and undemanding environment. The girls' narratives revealed that they experienced a sense of belonging when they participated in the JiT sessions, that they could be themselves and felt accepted for who they are. According to SDT, “relatedness” is one of three basic psychological needs to be fulfilled in order to enhance self-motivation, personal development and well-being across the lifespan. It refers to the need to feel accepted in one's social milieu (37). This indicates that the JiT intervention provided a context where the need for relatedness was met, which made it a safe foundation to engage in the new activity of combined dance and yoga and to be playful and creative together. This may also be an explanatory mechanism for other positive experiences and effects of the intervention. The girls who were interviewed described dance and yoga as fun physical activities; they observed that dance brought a sense of happiness while yoga, especially the relaxation element, facilitated calmness. These experiences show that both activities influenced the girls' minds and emotions, which is what the theory of embodiment is about (39, 40). One girl explained that dancing made her stronger and therefore it felt better inside. Evidence suggests that the connection between movement and emotion is bidirectional, in other words, by consciously choosing postures and movements our feelings can be affected (47–49). Developing the capacity to choose movements can therefore empower embodiment skills, which have the potential to strengthen emotional resiliency and self-regulation (49). Girls who participated in the JiT intervention felt more self-confident after the intervention. This is in line with Schwender et al. (29), who conclude from qualitative studies that dance interventions can strengthen self-trust, self-esteem and self-expression among children and adolescents. The girls experienced relief from abdominal pain and disturbing thoughts at the JiT sessions and subsequently they experienced less pain in their daily life and felt calmer. When the girls learned at the JiT sessions about how they could use their body to regulate emotions and pain, this strengthened their own ability to affect their situation, which facilitated increased participation in activities. For example, they increased their attendance in school, which is also in accordance with results shown by Korterink et al. (25). According to SDT, this could be viewed as satisfying another of the three basic needs, competence, in other words, being able to make a positive change for a desired outcome (38). The ability to flexibly control emotions, direct attention and change perspectives has proven to be an important component of health outcomes in children and adolescents (50).

The relief from abdominal pain that the girls expressed in this study is consistent with the results from our previous quantitative study that showed a reduction of abdominal pain among girls who participated in the JiT dance and yoga intervention compared to controls (32). Moreover, it is in line with other intervention studies on yoga (25, 51) and cognitive behavioural therapy (52–58) that have shown a reduction in pain frequencies (25, 51–58) and pain intensity (25, 52–55, 57, 58) among children and adolescents with FAPDs. A qualitative analysis of an internet-based psychosocial intervention (rooted in the cognitive-behavioural model), designed specifically for children with FAP and their parents (56) revealed that the children highlighted strategies such as relaxation (progressive muscle relaxation and breathing techniques) and distraction techniques (attention focusing, imagination and mental games) as successful coping mechanisms. This is in line with results from this study, since the participants perceived pain reduction, learned exercises and used them in different situations in their daily lives to reduce and manage pain. Evans et al. (59) found, in their qualitative analysis of a yoga intervention, that adolescents with IBS reported experiencing abdominal-opening poses, for instance the bridge pose, and other inversions, as helpful for the abdomen and that yoga felt comfortable, which partly agrees with how the girls in our study experienced yoga. The JiT intervention was 8 months long and of course many things happened in the girls' lives during this period; thus, it is not possible to know to what extent their participation in the JiT intervention influenced their lives and how much was the result of natural development or other events in their lives. Previous qualitative research (60) has shed light on how children with FAPDs experience living with the disorder, revealing that they often missed days in school and found it hard to focus in school. They felt a need for strategies to manage the abdominal pain; furthermore, they found that focusing on positive thoughts and activities helped to relieve the pain. This study contributes with new insights into how a combined dance and yoga intervention can meet those needs by providing a positive activity in a safe context, which can ease pain and increase participation in daily activities.

### 4.1. Methodological considerations

All authors were actively involved in the analysis process and discussed the steps in the analysis several times, which strengthens credibility (61). Presenting quotations in the result also strengthens credibility (61) and all authors agreed on the accuracy of the quotations. Two of the authors (SH and AD) had a closer involvement in the intervention and two (ME and EM) had a more distant role in the research team, which we define as a strength Moreover, the authors had different backgrounds in the medical field and previous experiences of qualitative research, as described in the supplemental file “Author information”, which further contributes to credibility (62, 63).

The fact that there were several interviewers with different experiences involved in the study might be both a strength and a weakness. It could be argued a strength that the first author, SH, who was also an instructor for three of the five intervention groups, did not conduct the interviews, which might have decreased the risk of participants being reluctant to be critical about the intervention. It is a possible weakness that there was more than one interviewer, since their previous experience and individual differences might affect the interviews, but they all used the same interview guide to strengthen dependability as much as possible. The girls were familiar with the interviewers from information meetings and from the quantitative data collection, and two of the interviewers had stood in as instructors a few times, which was a strength in building a trusting atmosphere during the interviews, since they were not complete strangers to the girls. There is still a risk that the participants felt more drawn to answer in a positive way, but to further decrease that risk they were encouraged to speak freely about their experiences, both negative and positive (45, 64). The 25 girls who were interviewed had all participated regularly during the eight-month intervention, which might increase the likelihood that they would have positive experiences from the intervention compared to those who dropped out from the intervention sessions earlier. To get a more complete picture, it would have been good to include participants who choose to quit participation in the intervention after some time and/or those who had a very low participation rate.

## 5. Conclusion

In conclusion, regular participation in an eight-month intervention with combined dance and yoga, in a supportive and non-judgemental atmosphere, can ease pain and strengthen inner resources, resulting in empowerment, well-being, and a more active life for girls with functional abdominal pain disorders.

## Data Availability

The datasets presented in this article are not readily available because of ethical restrictions due to the personal nature of the interviews. Requests to access the datasets should be directed to sofie.hogstrom@oru.se.
